# Invisible exposure, visible harm: air pollution and the global burden of cardiovascular disease

**DOI:** 10.1186/s41021-026-00366-y

**Published:** 2026-11-10

**Authors:** Neha Thakur, Hrishita Biswas, Arpita Das, Sneha Thakur, Rajgourab Ghosh

**Affiliations:** 1 Amity Institute of Biotechnology, Amity University Kolkata, Kolkata, West Bengal 700135 India; 2 Amity Institute of Dietetics and Nutrition, Amity University Kolkata, Kolkata, West Bengal 700135 India

**Keywords:** Air pollution, Particulate matter, Cardiovascular disease, Oxidative stress, Endothelial dysfunction, Public health policy

## Abstract

**Background:**

Air pollution is a major modifiable environmental risk factor for cardiovascular disease (CVD). In the Global Burden of Disease (GBD) 2021 study, air pollution was the second-leading risk factor for death worldwide, accounting for an estimated 8.1 million deaths from all causes in 2021, and particulate matter (PM) pollution (ambient plus household) was associated with approximately 4.5 million cardiovascular deaths.

**Main body:**

This narrative review, based on a structured search of PubMed/MEDLINE, Scopus and Web of Science, summarizes evidence on the sources of the main pollutants (fine particulate matter [PM₂.₅], coarse particulate matter [PM₁₀], ultrafine particles [PM₀.₁/UFP], nitrogen dioxide [NO₂], sulfur dioxide [SO₂], ozone [O₃] and carbon monoxide [CO]); the global burden of pollution-attributable CVD; epidemiological evidence from major cohort and multi-city studies; biological mechanisms, distinguishing those supported by human data from those derived mainly from cell and animal models; the associations with specific cardiovascular phenotypes (ischemic heart disease, stroke, hypertension, heart failure, arrhythmia and vascular disease); susceptible populations; transcriptomic, proteomic, lipidomic, epigenetic and microRNA findings; and preventive strategies graded by strength of evidence. Observational studies consistently associate short- and long-term exposure to PM₂.₅ with cardiovascular events and mortality, and controlled-exposure, quasi-experimental and mechanistic studies support a causal interpretation for PM₂.₅. Evidence for individual gaseous pollutants is less consistent, and most multi-omics findings remain exploratory.

**Conclusion:**

PM₂.₅ should be regarded as a cardiovascular risk factor in clinical and public-health practice. Key uncertainties include the shape of the exposure–response relationship at very low concentrations, the relative toxicity of particle components, and whether reducing individual exposure lowers hard cardiovascular outcomes. Priorities include prospective multi-omics studies with clinical endpoints in low- and middle-income countries, causal-inference designs, and trials testing exposure-reduction and targeted therapeutic strategies in highly exposed populations.

## Background

Rapid industrialization, urbanization, growth in motorized transport and continued reliance on fossil fuels and solid-fuel combustion have made air pollution one of the most important environmental threats to health [1, 2]. Although air pollution has long been recognized as a cause of respiratory disease, a large proportion of the deaths attributed to it are cardiovascular [3–5].

Cardiovascular disease remains the leading cause of death worldwide, with an estimated 19.8 million deaths in 2022 [6]. Beyond the traditional risk factors (hypertension, dyslipidemia, diabetes, smoking, diet and physical inactivity), there is now strong evidence that air pollution is an independent cardiovascular risk factor [3, 7, 8]. Reflecting this evidence, in 2021 the World Health Organization (WHO) lowered its guideline value for annual mean PM₂.₅ from 10 µg/m³ (the 2005 guideline) to 5 µg/m³ [9, 10]. Both short-term (hours to days) and long-term (months to years) exposures are associated with higher rates of hospitalization, acute cardiovascular events and premature death [11, 12].

Pollutants differ in their dominant biological effects. PM₂.₅ and ultrafine particles penetrate deep into the lung and may promote oxidative stress, inflammation, reduced nitric oxide bioavailability and vascular dysfunction [3, 13]. NO₂ and SO₂ act mainly as airway irritants but may also affect autonomic reflexes; O₃ is a strong photochemical oxidant; and CO impairs oxygen delivery and can provoke myocardial ischemia, especially in people with existing coronary disease [7]. Older adults, people with pre-existing cardiovascular or metabolic disease and socioeconomically disadvantaged groups appear to be most affected [3, 14].

Much less is known about the molecular determinants of individual susceptibility. Transcriptomic, proteomic, lipidomic, epigenetic and microRNA (miRNA) studies may help identify biological pathways and candidate biomarkers of exposure and effect, but most of these findings are preliminary [15]. Combined with high-resolution exposure modelling and precision-medicine approaches, they could eventually improve risk prediction; this potential has not yet been tested in studies with clinical endpoints.

This review summarizes current evidence on (1) the classification and sources of major air pollutants; (2) the global burden of pollution-attributable CVD; (3) epidemiological evidence from major cohort and multi-city studies; (4) biological mechanisms linking exposure to cardiovascular injury; (5) associations with specific cardiovascular phenotypes; (6) susceptible populations; (7) multi-omics findings; and (8) preventive strategies, graded by strength of evidence. We conclude with current knowledge gaps and priorities for future research.

## Methods: literature search and selection

This is a narrative review informed by a structured literature search. PubMed/MEDLINE, Scopus and Web of Science were searched for articles published between January 2000 and August 2026 (final search: September 2026); Google Scholar and the reference lists of key reviews, scientific statements and guidelines were screened for additional records. Landmark studies published before 2000 (e.g., the Harvard Six Cities Study) were included when they remained central to the evidence base.

Search strings combined exposure terms (“air pollution”, “particulate matter”, “PM2.5”, “PM10”, “ultrafine particles”, “nitrogen dioxide”, “sulfur dioxide”, “ozone”, “carbon monoxide”, “household air pollution”, “solid fuel”) with outcome terms (“cardiovascular disease”, “ischemic heart disease”, “myocardial infarction”, “stroke”, “hypertension”, “heart failure”, “arrhythmia”, “atrial fibrillation”, “atherosclerosis”, “cardiovascular mortality”) and, for the mechanistic sections, with terms such as “oxidative stress”, “inflammation”, “endothelial dysfunction”, “autonomic”, “NF-κB”, “Nrf2”, “NLRP3”, “transcriptome”, “proteome”, “lipidome”, “DNA methylation”, “microRNA” and “Mendelian randomization”.

We included peer-reviewed English-language original studies, systematic reviews/meta-analyses and scientific statements, as well as reports from WHO, the GBD collaboration, the Health Effects Institute (HEI) and the U.S. Environmental Protection Agency (EPA). For epidemiological statements we prioritized large prospective cohorts, multi-city time-series studies and meta-analyses; for burden estimates, GBD and WHO sources; and for mechanisms, controlled human exposure and panel studies, with animal and in-vitro studies used as supporting evidence and identified as such. Conference abstracts, non-peer-reviewed material (other than agency reports) and studies without a cardiovascular endpoint or intermediate cardiovascular marker were excluded. Because this is a narrative review, no formal risk-of-bias assessment or quantitative pooling was performed; the strength of evidence is described qualitatively.

## Classification and sources of air pollutants

Air pollution is usually classified by the setting of exposure as ambient (outdoor) or household (indoor). Ambient pollution comes mainly from road traffic, power generation, industry, agriculture and open burning, with natural contributions from dust storms, wildfires and volcanic activity. Household air pollution in low- and middle-income countries (LMICs) is dominated by the burning of solid fuels (wood, crop residues, dung, charcoal and coal) for cooking and heating; other indoor sources include tobacco smoke, incense, candles, gas stoves and volatile organic products [2, 16].

For regulatory purposes, the U.S. EPA distinguishes six “criteria” pollutants for which National Ambient Air Quality Standards are set — PM (PM₁₀ and PM₂.₅), O₃, CO, SO₂, NO₂ and lead (Pb) — from hazardous air pollutants, such as benzene, polycyclic aromatic hydrocarbons (PAHs), dioxins and toxic metals, which are regulated because of their carcinogenic or other serious health effects [17]. Pollutants may also be classified as primary (emitted directly, e.g., CO, SO₂, nitrogen oxides and primary PM) or secondary (formed in the atmosphere, e.g., O₃ and secondary sulfate, nitrate and organic aerosols), and as gaseous or particulate [2].

Particles are classified by aerodynamic diameter as PM₁₀ (≤ 10 μm, including the coarse fraction 2.5–10 μm), PM₂.₅ (≤ 2.5 μm; fine particles) and PM₀.₁ (≤ 0.1 μm; ultrafine particles, UFP). Particle composition — elemental and organic carbon, secondary inorganic ions (sulfate, nitrate, ammonium), transition metals and semi-volatile organic compounds such as PAHs — varies by source and determines the oxidative potential of the particles [18, 19]. Figure 1 summarizes these classifications, and Table 1 lists the main sources, current WHO guideline values and principal cardiovascular associations of each pollutant.

**Fig. 1:**
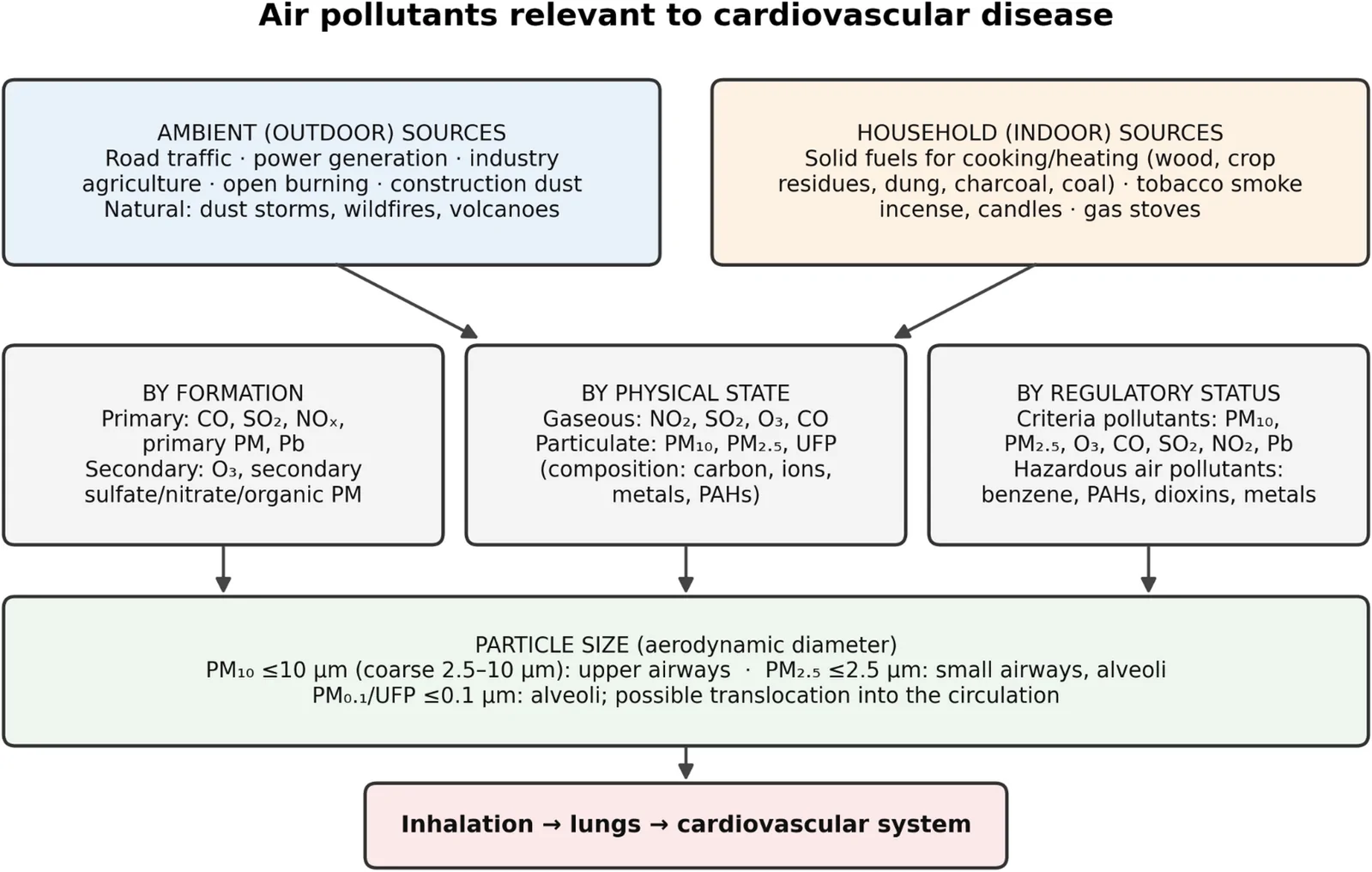
Classification and sources of the major air pollutants relevant to cardiovascular disease. Pollutants are grouped by setting of exposure (ambient vs. household), by formation (primary vs. secondary) and by physical state; particles are further classified by aerodynamic diameter. Based on [2, 9, 17]

**Table 1:** Major air pollutants: main sources, WHO 2021 air quality guideline (AQG) levels and principal cardiovascular associations

Pollutant	Main sources	WHO 2021 AQG	Principal cardiovascular associations (effect estimates)	Ref.
PM₂.₅	Road traffic, power generation, industry, residential solid-fuel burning, wildfires; secondary aerosol formation	Annual 5 µg/m³; 24-h 15 µg/m³	Long-term: CVD mortality RR 1.11 (95% CI 1.09–1.14), IHD mortality RR 1.16 (1.10–1.21) and stroke mortality RR 1.11 (1.04–1.18) per 10 µg/m³. Short-term: MI RR 1.025 (1.015–1.036), HF hospitalization/death + 2.12% (1.42–2.82%) and daily CV mortality + 0.55% (0.45–0.66%) per 10 µg/m³	[11, 12, 20, 21]
PM₁₀	Road and construction dust, mining, agriculture, industry, combustion	Annual 15 µg/m³; 24-h 45 µg/m³	Short-term: MI RR 1.006 (1.002–1.009) and HF hospitalization/death + 1.63% (1.20–2.07%) per 10 µg/m³. Long-term: acute coronary events HR 1.12 (1.01–1.25) per 10 µg/m³ (ESCAPE)	[20–22]
UFP (PM₀.₁)	Fresh combustion emissions (traffic, aviation), nucleation of vapours	No numerical AQG (good-practice statements only)	Evidence mainly from panel and controlled-exposure studies (vascular dysfunction, impaired fibrinolysis); long-term epidemiological evidence limited and inconsistent	[9, 23, 24]
NO₂	Road traffic (especially diesel), power generation, industry	Annual 10 µg/m³; 24-h 25 µg/m³	Short-term: HF hospitalization/death + 1.70% (1.25–2.16%) per 10 ppb; stroke RR 1.014 (1.009–1.019) per 10 ppb; MI RR 1.011 (1.006–1.016) per 10 µg/m³. Long-term: higher odds of hypertension	[20, 21, 25, 26]
SO₂	Combustion of sulfur-containing coal and oil (power plants, industry, shipping), metal smelting	24-h 40 µg/m³	Short-term: HF hospitalization/death + 2.36% (1.35–3.38%) per 10 ppb; stroke RR 1.019 (1.011–1.027) per 10 ppb; MI RR 1.010 (1.003–1.017) per 10 µg/m³; associated with hypertension in meta-analysis. Effects difficult to separate from co-emitted PM	[20, 21, 25, 26]
O₃	Secondary pollutant formed from NOₓ and volatile organic compounds in sunlight	Peak season 60 µg/m³; 8-h 100 µg/m³	Short-term associations with MI and HF not statistically significant in meta-analyses; long-term all-cause mortality HR 1.011 (1.010–1.012) per 10 ppb in U.S. Medicare cohort. Cardiovascular evidence less consistent than for PM₂.₅	[20, 21, 27]
CO	Incomplete combustion: vehicle exhaust, biomass and solid-fuel burning	24-h 4 mg/m³	Short-term: MI RR 1.048 (1.026–1.070) per 1 mg/m³; HF hospitalization/death + 3.52% (2.52–4.54%) per 1 ppm; reduced oxygen delivery and myocardial ischemia	[7, 20, 21]
Lead (Pb)	Historical leaded fuel, smelting, battery recycling, industrial emissions	Not covered by WHO 2021 AQG	Blood lead concentration (1.0 vs. 6.7 µg/dL) associated with CVD mortality HR 1.70 (1.30–2.22) in U.S. adults; hypertension	[28]

AQG, air quality guideline; CI, confidence interval; CV, cardiovascular; CVD, cardiovascular disease; HF, heart failure; HR, hazard ratio; IHD, ischemic heart disease; MI, myocardial infarction; RR, relative risk; UFP, ultrafine particles. Effect estimates are pooled estimates from the cited meta-analyses or cohort studies and are associations from observational studies

## Global burden of air pollution-attributable cardiovascular disease

According to WHO, 99% of the world’s population lived in places where the 2021 air quality guideline levels were not met [29]. Estimates of the resulting health burden differ according to the pollutants included, the exposure–response functions and counterfactual concentrations used, the outcomes considered and the reference year. Three quantities should therefore be distinguished (Table 2): (a) deaths from all causes attributable to air pollution; (b) cardiovascular deaths attributable to air pollution; and (c) the separate contributions of ambient and household pollution.

a **All-cause deaths.** GBD 2019 estimated that air pollution was responsible for 6.67 million (95% uncertainty interval [UI] 5.90–7.49 million) deaths in 2019 [30, 31]. In GBD 2021, air pollution (ambient PM₂.₅, household PM₂.₅ from solid fuels and ambient O₃) was the second-leading risk factor for death after high blood pressure, with 8.1 million attributable deaths in 2021, and PM pollution was the leading risk factor for disability-adjusted life-years (DALYs) globally [32, 33]. Because each GBD round re-estimates the whole time series with updated data and methods, estimates from different rounds should not be compared directly. Models based on the Global Exposure Mortality Model (GEMM), which assume steeper risks at high concentrations and include all non-accidental deaths, give higher totals: 8.9 million deaths from ambient PM₂.₅ in 2015 [34] and 8.8 million excess deaths from ambient PM₂.₅ and O₃ in 2015 [35]. A source-specific analysis attributed 10.2 million deaths in 2012 to PM₂.₅ from fossil-fuel combustion alone [36].
b. **Cardiovascular deaths.** In GBD, O₃ is linked only to chronic obstructive pulmonary disease, so all air pollution-attributable cardiovascular deaths come from PM pollution. Using GBD 2021 data, PM pollution (ambient plus household) was associated with approximately 4.48 million cardiovascular deaths and 99.6 million cardiovascular DALYs in 2021, with age-standardized rates inversely related to the socio-demographic index (SDI) [5]. Thus, more than half of all air pollution-attributable deaths are cardiovascular, and roughly one in five cardiovascular deaths worldwide is attributable to PM pollution [5, 6].
c. **Ambient versus household pollution.** In GBD 2019, ambient PM pollution accounted for 4.14 million (95% UI 3.45–4.80 million) deaths and household air pollution from solid fuels for 2.31 million (1.63–3.12 million) deaths [30]. These components are not strictly additive, because GBD estimates the joint burden of overlapping exposures. Although deaths from household air pollution have declined, the burden from ambient PM pollution has increased, and both remain concentrated in South and East Asia and sub-Saharan Africa [30, 31].

Heart failure (HF) is an important but less frequently quantified consequence. Using GBD 2021 data, Dong et al. estimated that 45.56 million people were living with CVD-related HF in 2021, with rising age-standardized prevalence (particularly in middle and high-middle SDI countries) and further increases projected to 2050; hypertensive heart disease and ischemic heart disease were the predominant causes [37]. Because both of these conditions, and HF hospitalization itself, are associated with PM exposure [11, 21], a pollution-related component is likely to contribute to the projected growth of HF in rapidly industrializing LMICs. However, that study did not estimate the HF burden attributable specifically to air pollution, and such estimates are still needed.

**Table 2:** Selected estimates of the global mortality burden attributable to air pollution and how they differ

Source	Reference year	Exposure(s)	Outcome	Estimate	Notes on methods
GBD 2019 [30]	2019	Ambient PM₂.₅ + household PM₂.₅ + O₃	All-cause deaths	6.67 million (95% UI 5.90–7.49)	Comparative risk assessment; MR-BRT exposure–response curves
GBD 2019 [30]	2019	Ambient PM₂.₅; household PM₂.₅	All-cause deaths	4.14 million (3.45–4.80); 2.31 million (1.63–3.12)	Components not strictly additive
GBD 2021 / State of Global Air 2024 [32, 33]	2021	Ambient PM₂.₅ + household PM₂.₅ + O₃	All-cause deaths	8.1 million	Second-leading risk factor for death; O₃ ≈ 489,000 deaths
GBD 2021 (He et al.) [5]	2021	PM pollution (ambient + household)	CVD deaths; CVD DALYs	4.48 million; 99.6 million DALYs	Inverse gradient with SDI
Burnett et al. (GEMM) [34]	2015	Ambient PM₂.₅	Non-accidental deaths	8.9 million	Hazard ratios from 41 cohorts in 16 countries
Lelieveld et al. [35]	2015	Ambient PM₂.₅ + O₃	Excess deaths	8.8 million	GEMM-based; mean loss of life expectancy 2.9 years
Vohra et al. [36]	2012	Fossil-fuel-derived PM₂.₅	Excess deaths	10.2 million	Chemical transport model (GEOS-Chem) with source attribution

CVD, cardiovascular disease; DALYs, disability-adjusted life-years; GBD, Global Burden of Disease; GEMM, Global Exposure Mortality Model; MR-BRT, meta-regression–Bayesian, regularized, trimmed; O₃, ozone; PM, particulate matter; SDI, socio-demographic index; UI, uncertainty interval. Estimates differ because of differences in exposure–response functions, counterfactual (minimum-risk) concentrations, outcomes included and reference year

## Particulate matter: from exposure to cardiovascular outcomes

### Particle characteristics and deposition

The cardiovascular relevance of PM depends on particle size, number, surface area and composition. Coarse particles are deposited mainly in the upper airways, whereas PM₂.₅ reaches the small airways and alveoli, and UFP are deposited efficiently in the alveolar region, where clearance is slower [38]. Because of their large surface area, UFP carry relatively large amounts of redox-active metals and organic compounds [18]. The oxidative potential of PM — its capacity to generate reactive oxygen species (ROS) — differs by source and may be more relevant to health than mass concentration alone [19].

### Exposure–response relationship and the question of a threshold

Meta-analyses of long-term cohort studies report that each 10 µg/m³ increase in PM₂.₅ is associated with an 8% increase in all-cause mortality and an 11% increase in cardiovascular mortality [11]. The exposure–response relationship is non-linear: the increase in risk per unit of exposure is steepest at low-to-moderate concentrations and flattens at very high concentrations [3, 34]. The integrated exposure–response function used in earlier GBD rounds combined PM₂.₅ from ambient air, household air, second-hand smoke and active smoking; subsequent rounds incorporated newer cohort data from low-exposure settings [30].

Current evidence has not identified a concentration below which PM₂.₅ has no cardiovascular effect. Associations persist in cohorts in which most exposures are below current standards — for example, among 60.9 million U.S. Medicare beneficiaries, the association with mortality was stronger when analysis was restricted to annual concentrations below 12 µg/m³ [27] — and similar findings have been reported in Canada at very low concentrations [39]. On this basis, WHO concluded that health effects occur at concentrations below its previous guideline and lowered the annual guideline to 5 µg/m³ [9]. However, the absence of an observed threshold partly reflects limited statistical power and residual confounding at very low concentrations; it does not prove that no threshold exists. It is therefore more accurate to state that no safe threshold has been identified than that none exists.

### Effect modification by other exposures

Climate change may amplify the cardiovascular effects of PM: higher temperatures favor O₃ formation and increase the frequency of wildfires and dust storms. PM₂.₅ from wildfire smoke has been associated with cardiopulmonary outcomes in older adults, with effect estimates comparable to those for PM₂.₅ from other sources [1, 40].

## Epidemiological evidence from major cohort and multi-city studies

The first major evidence came from U.S. cohorts. In the Harvard Six Cities Study, mortality was 26% higher in the most polluted than in the least polluted city, with cardiopulmonary deaths accounting for much of the difference [41]. In extended follow-up, reductions in PM₂.₅ between study periods were associated with lower mortality [42]. In the American Cancer Society Cancer Prevention Study II (approximately 500,000 adults), long-term PM₂.₅ exposure was most strongly associated with ischemic heart disease (IHD) mortality [43]. In the Women’s Health Initiative, each 10 µg/m³ increase in PM₂.₅ was associated with a 24% higher risk of a first cardiovascular event and a 76% higher risk of cardiovascular death [44]. Among 60.9 million Medicare beneficiaries, associations with mortality were observed even at concentrations below the U.S. standard [27].

In Europe, the ESCAPE project pooled 11 cohorts with 100,166 participants and found that long-term PM exposure was associated with incident acute coronary events, including at concentrations below the European Union limit values [22]. Evidence from China, where concentrations are much higher, shows similar associations: a national cohort of 189,793 men reported higher cardiovascular mortality with increasing long-term PM₂.₅ exposure [45], and the China-PAR cohort of 116,972 adults reported a 25% higher risk of incident CVD per 10 µg/m³ [46]. Short-term associations have been replicated in a time-series study of 652 cities in 24 countries and regions [12] and in meta-analyses of myocardial infarction, stroke and HF [20, 21, 25]. An umbrella review of systematic reviews and meta-analyses has summarized the consistency of these associations across outcomes [47]. Key studies are summarized in Table 3.

**Table 3:** Characteristics and principal findings of major epidemiological studies of air pollution and cardiovascular outcomes

Study (design)	Population / setting	Sample size	Exposure	Follow-up	Cardiovascular endpoint	Principal effect estimate (95% CI)
Harvard Six Cities [41] (prospective cohort)	Adults, 6 U.S. cities	8,111	PM₂.₅ (between-city contrast)	14–16 years	All-cause and cardiopulmonary mortality	Mortality rate ratio 1.26 (1.08–1.47), most vs. least polluted city
Six Cities extended follow-up [42]	Same cohort	8,096	PM₂.₅ (city-specific, time-varying)	1974–1998	CV mortality; change in exposure	CV mortality RR 1.28 (1.13–1.44) per 10 µg/m³; total mortality RR 0.73 (0.57–0.95) per 10 µg/m³ decrease
ACS CPS-II [43] (prospective cohort)	U.S. adults	≈ 500,000	PM₂.₅ (metropolitan mean)	16 years (1982–1998)	IHD mortality	RR 1.18 (1.14–1.23) per 10 µg/m³
Women’s Health Initiative [44] (prospective cohort)	Post-menopausal women, 36 U.S. cities	65,893	PM₂.₅ (nearest monitor)	Median 6 years	First CV event; CV death	HR 1.24 (1.09–1.41) and HR 1.76 (1.25–2.47) per 10 µg/m³
ESCAPE [22] (11 pooled cohorts)	Adults in 5 European countries	100,166	PM₂.₅; PM₁₀ (land-use regression)	Mean 11.5 years	Incident acute coronary events	HR 1.13 (0.98–1.34) per 5 µg/m³ PM₂.₅; HR 1.12 (1.01–1.25) per 10 µg/m³ PM₁₀
MESA Air [48] (prospective cohort)	Adults free of CVD, 6 U.S. metropolitan areas	6,795	PM₂.₅; NOₓ (individual-level)	≈ 10 years	Coronary artery calcium progression	+ 4.1 Agatston units/year (1.4–6.8) per 5 µg/m³ PM₂.₅
U.S. Medicare [27] (open cohort)	Adults ≥ 65 years	60,925,443	PM₂.₅; O₃ (1-km model)	2000–2012	All-cause mortality	HR 1.073 (1.071–1.075) per 10 µg/m³; HR 1.136 (1.131–1.141) below 12 µg/m³
Chinese men [45] (prospective cohort)	Men ≥ 40 years, 45 areas of China	189,793	PM₂.₅ (satellite-based)	≈ 15 years	CVD mortality	HR 1.09 (1.08–1.10) per 10 µg/m³
China-PAR [46] (prospective cohort)	Adults in 15 Chinese provinces	116,972	PM₂.₅ (satellite-based)	≈ 8 years	Incident CVD; CVD mortality	HR 1.25 (1.22–1.28) and HR 1.16 (1.12–1.21) per 10 µg/m³
Chen & Hoek [11] (meta-analysis)	Cohorts worldwide	107 studies	Long-term PM₂.₅	—	CVD, IHD and stroke mortality	RR 1.11 (1.09–1.14), 1.16 (1.10–1.21) and 1.11 (1.04–1.18) per 10 µg/m³
MCC network [12] (multi-city time series)	652 cities, 24 countries/regions	≈ 60 million deaths	Short-term PM₂.₅ (2-day mean)	1986–2015	Daily CV mortality	+ 0.55% (0.45–0.66%) per 10 µg/m³
Mustafic et al. [20] (meta-analysis)	Worldwide	34 studies	Short-term PM₂.₅, PM₁₀, NO₂, SO₂, CO, O₃	—	Myocardial infarction	PM₂.₅ RR 1.025 (1.015–1.036) per 10 µg/m³; CO RR 1.048 (1.026–1.070) per 1 mg/m³
Shah et al. [21] (meta-analysis)	Worldwide	35 studies	Short-term PM and gases	—	HF hospitalization or death	PM₂.₅ +2.12% (1.42–2.82%) per 10 µg/m³
Shah et al. [25] (meta-analysis)	28 countries	103 studies	Short-term PM and gases	—	Stroke hospitalization or death	PM₂.₅ RR 1.011 (1.011–1.012) per 10 µg/m³

ACS CPS-II, American Cancer Society Cancer Prevention Study II; China-PAR, Prediction for Atherosclerotic Cardiovascular Disease Risk in China; CV, cardiovascular; CVD, cardiovascular disease; ESCAPE, European Study of Cohorts for Air Pollution Effects; HF, heart failure; HR, hazard ratio; IHD, ischemic heart disease; MCC, Multi-Country Multi-City Collaborative Research Network; MESA, Multi-Ethnic Study of Atherosclerosis; NOₓ, nitrogen oxides; RR, relative risk. All estimates are adjusted associations from observational studies

### From association to causation

Most of the evidence above is observational, and associations do not by themselves establish causation; residual confounding by socioeconomic position, co-pollutants, noise and temperature, as well as exposure misclassification, can bias estimates in either direction. The causal interpretation for PM₂.₅ rests on the convergence of several lines of evidence: consistency across populations, study designs and exposure ranges; temporality and exposure–response gradients; controlled human exposure studies showing acute vascular and thrombotic effects [23, 48, 49]; experimental animal studies [50]; and quasi-experimental studies in which reductions in pollution were followed by improvements in life expectancy or mortality [42, 51, 52]. Not all such natural experiments are conclusive — for example, re-analysis of the Irish coal bans found clear reductions in respiratory mortality but weaker evidence for cardiovascular mortality once secular trends were accounted for [53]. On the basis of the totality of evidence, the American Heart Association and the U.S. EPA have concluded that the relationship between PM₂.₅ exposure and cardiovascular effects is causal [3, 54]. For individual gaseous pollutants, the evidence is weaker and more susceptible to confounding by co-emitted particles, and associations should be interpreted as such.

## Mechanisms of cardiovascular injury

Three broad pathways have been proposed to link inhaled pollutants with cardiovascular injury (Fig. 2) [1, 3, 8]: (i) pulmonary oxidative stress and inflammation, with “spill-over” of inflammatory mediators into the systemic circulation; (ii) activation of pulmonary sensory receptors leading to autonomic imbalance; and (iii) translocation of ultrafine particles or soluble particle constituents into the circulation. These pathways converge on endothelial dysfunction, vasoconstriction and increased blood pressure, a prothrombotic state and progression and destabilization of atherosclerotic plaque. Table 4 summarizes the strength of human and experimental evidence for each mechanism.

**Fig. 2:**
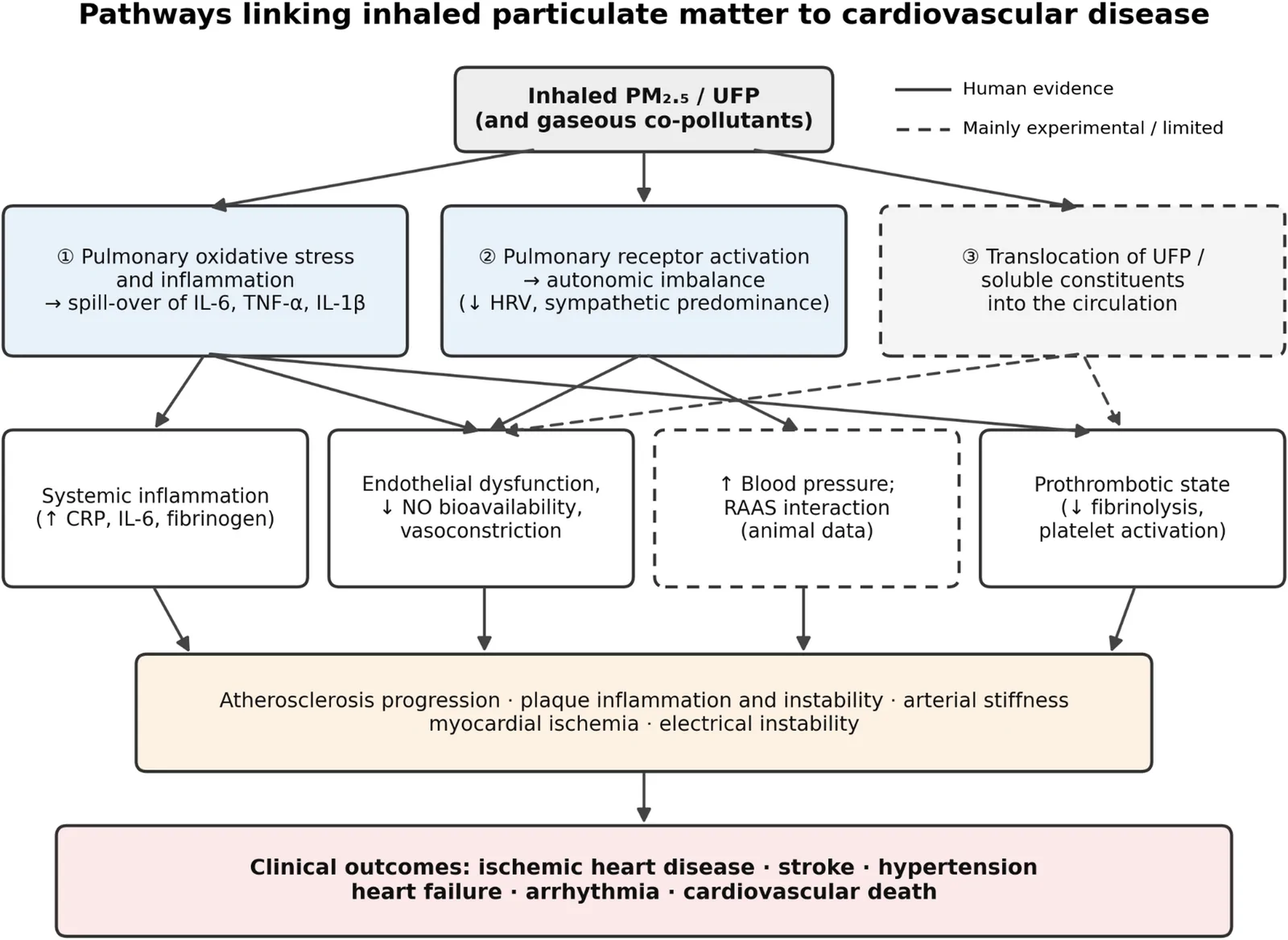
Integrated pathways linking inhaled particulate matter to cardiovascular disease. Solid borders indicate mechanisms supported by human evidence (controlled exposure, panel or cohort studies); dashed borders indicate mechanisms supported mainly by animal or cell studies. Based on [1, 3, 8, 24]

### Pulmonary inflammation, systemic inflammation and oxidative stress

When particles are deposited in the lung, alveolar macrophages and epithelial cells release pro-inflammatory mediators, including interleukin (IL)-1β, IL-6, IL-8 and tumor necrosis factor-α (TNF-α) [55]. In humans, short- and long-term PM exposures are associated with higher circulating C-reactive protein (CRP), IL-6 and fibrinogen [56, 57], and episodes of higher PM₂.₅ have been associated with markers of endothelial injury and systemic inflammation [58]. In rodents, inhaled PM rapidly increases ROS in the lung and heart [59], and chronic exposure causes vascular dysfunction through NADPH oxidase- and Toll-like receptor 4 (TLR4)-dependent pathways [60].

These inflammatory markers are relevant to hypertension, endothelial dysfunction and cardiovascular remodeling. As summarized in the recent review by Tsioufis et al., elevated CRP, IL-6 and TNF-α in hypertension are associated with endothelial dysfunction, arterial stiffness and target-organ damage [61]. Mechanistically, IL-6 drives hepatic production of CRP and fibrinogen, and TNF-α and IL-6 reduce endothelial nitric oxide bioavailability and promote vascular smooth-muscle proliferation and fibrosis [61, 62]. A pollution-induced low-grade inflammatory state may therefore contribute to both the development of hypertension and structural vascular and cardiac remodeling. However, not every biomarker is a causal mediator: Mendelian randomization (MR) studies indicate that CRP is unlikely to cause coronary heart disease, whereas IL-6 receptor signaling probably does [63, 64]. Accordingly, CRP should be viewed as a marker of pollution-related inflammation, and IL-6 as a candidate mediator.

### Autonomic imbalance and blood pressure

Activation of pulmonary neural receptors by particles and irritant gases can shift autonomic balance toward sympathetic predominance. In a meta-analysis of panel studies, higher PM exposure was associated with reduced heart-rate variability (HRV) [65, 66]. Sympathetic activation increases vascular tone, heart rate and myocardial oxygen demand and may lower the threshold for arrhythmia. In controlled human exposure studies, inhalation of concentrated ambient particles plus O₃ caused acute brachial artery vasoconstriction [48]. In animal models, PM exposure potentiated angiotensin II-induced hypertension through ROS-mediated activation of the Rho/ROCK pathway, suggesting interaction with the renin–angiotensin–aldosterone system (RAAS) [67].

### Endothelial dysfunction, thrombosis and atherosclerosis

In randomized controlled exposure studies, dilute diesel exhaust impaired endothelium-dependent vasodilation and reduced acute release of tissue plasminogen activator in healthy volunteers [23], and increased exercise-induced myocardial ischemia in men with stable coronary heart disease [49]. Experimental studies show that PM promotes coagulation through factor XII activation [68] and accelerates atherosclerosis and vascular inflammation in apolipoprotein E-deficient mice [50]. In humans, long-term PM₂.₅ exposure is associated with faster progression of coronary artery calcification [69, 70].

### Particle translocation

Whether particles themselves enter the circulation in humans has been debated. An early study suggested rapid passage of inhaled technetium-labelled carbon particles into the blood [71], but a subsequent study found no significant translocation of carbon nanoparticles [72]. More recently, inhaled gold nanoparticles were detected in blood and urine of healthy volunteers and accumulated at sites of vascular inflammation in mice and in human surgical specimens [24]. Direct translocation is therefore plausible for UFP, but its quantitative contribution relative to the inflammatory and autonomic pathways is uncertain.

### Molecular signaling pathways

Much of the molecular evidence comes from cell and animal studies and should be interpreted accordingly (Fig. 3; Table 4). ROS generated by particle constituents activate nuclear factor-κB (NF-κB), which induces cytokines (IL-1β, IL-6, TNF-α), adhesion molecules (ICAM-1, VCAM-1, E-selectin) and chemokines that recruit monocytes to the vessel wall [8, 62]. The nuclear factor erythroid 2-related factor 2 (Nrf2)–Keap1 system is the main antioxidant counter-regulatory pathway, inducing heme oxygenase-1 (HO-1), NAD(P)H quinone oxidoreductase 1 and glutathione S-transferases; genetic variation in antioxidant pathways has been proposed to modify susceptibility, although human evidence is limited [15]. Mitogen-activated protein kinases (ERK1/2, JNK, p38) and phosphoinositide 3-kinase/Akt signaling have been implicated in vascular smooth-muscle proliferation, endothelial apoptosis and insulin resistance in experimental models [1, 62]. PM activates the NLRP3 inflammasome in macrophages, leading to caspase-1-dependent maturation of IL-1β and IL-18, and TLR4 and downstream JAK/STAT signaling have been implicated in pollution-induced vascular inflammation in mice [60, 62]. Mitochondria are both a source and a target of PM-induced ROS, and mitochondrial dysfunction has been reported in cardiac tissue of exposed animals [62]. These pathways provide biological plausibility, but their relative importance in humans exposed at ambient concentrations has not been established.

**Fig. 3:**
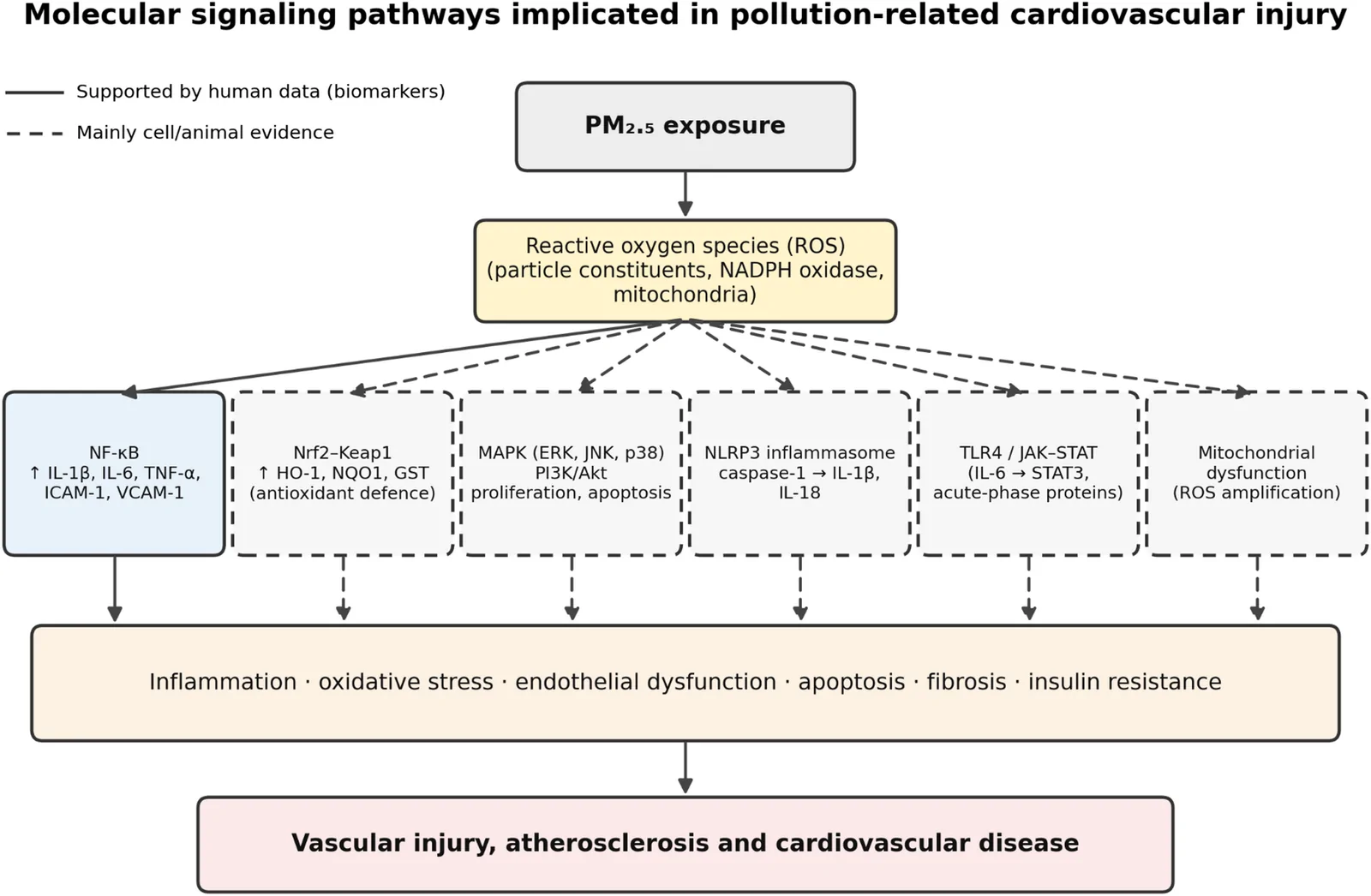
Principal molecular signaling pathways implicated in air pollution-related cardiovascular injury. Reactive oxygen species (ROS) are a common upstream signal. Evidence for most pathways comes predominantly from cell and animal models; human evidence is indicated where available. Based on [8, 15, 60, 62]

**Table 4:** Mechanisms linking air pollution to cardiovascular disease: evidence in humans and experimental models

Mechanism	Human evidence	Experimental (animal/cell) evidence	Overall strength in humans	Clinical relevance
Systemic inflammation (CRP, IL-6, fibrinogen)	Consistent associations in panel and cohort studies and meta-analysis [57, 58, 59]	Cytokine release from macrophages and epithelial cells [56]	Strong for association; causal role of individual markers uncertain (CRP probably not causal; IL-6 probably causal) [64, 65]	Hypertension, atherosclerosis, remodeling [62]
Autonomic imbalance	Reduced HRV in meta-analysis of panel studies [66]	Pulmonary reflex activation	Moderate	Arrhythmia, BP elevation
Endothelial dysfunction / vasoconstriction	Randomized controlled exposures [23, 49]	NADPH oxidase/TLR4-dependent vascular dysfunction [61]	Strong (acute effects)	Hypertension, ischemia
Prothrombotic state	Impaired fibrinolysis in controlled exposure [23, 50]	Factor XII activation [69]	Moderate	Acute coronary syndromes, stroke
Atherosclerosis progression	Coronary calcium progression in cohort [48]	Accelerated plaque in ApoE⁻/⁻ mice [51]	Moderate	IHD, stroke
Particle translocation	Conflicting; gold nanoparticles detected in blood [24, 71, 72]	Accumulation in vascular lesions [24]	Limited	Uncertain
RAAS activation	Indirect (BP associations)	PM potentiates angiotensin II hypertension via Rho/ROCK [68]	Limited	Hypertension, resistant hypertension (hypothesis)
NF-κB, Nrf2, MAPK, PI3K/Akt, NLRP3, JAK/STAT, mitochondria	Largely indirect (biomarkers, gene expression)	Extensive [8, 63]	Limited	Candidate targets (not validated)

ApoE⁻/⁻, apolipoprotein E-deficient; BP, blood pressure; CRP, C-reactive protein; HRV, heart-rate variability; IHD, ischemic heart disease; IL, interleukin; MAPK, mitogen-activated protein kinase; NF-κB, nuclear factor-κB; Nrf2, nuclear factor erythroid 2-related factor 2; RAAS, renin–angiotensin–aldosterone system; TLR4, Toll-like receptor 4

### Strengthening causal inference: Mendelian randomization

Mendelian Randomization uses genetic variants as instrumental variables to estimate causal effects that are less susceptible to confounding and reverse causation than conventional observational analyses. For example, a two-sample MR study using genome-wide association data from 3.38 million individuals and arterial stiffness data from 127,121 UK Biobank participants found that genetic liability to smoking initiation — another inhaled combustion exposure — was associated with higher arterial stiffness, whereas alcohol consumption was not [73]. Ambient pollution itself cannot be instrumented genetically, but MR can be applied to the intermediate pathways proposed here (e.g., IL-6 signaling, oxidative-stress and antioxidant pathways, blood pressure and lipids) and, in gene–environment interaction designs, to test whether genetically determined susceptibility modifies the effect of pollution on vascular phenotypes. Such designs would complement quasi-experimental studies and help to distinguish causal mediators from markers [63, 64].

## Air pollution across major cardiovascular phenotypes

Although most research has focused on IHD, air pollution is associated with a broad range of cardiovascular phenotypes. The evidence for each is summarized below; effect estimates are from observational studies unless stated otherwise.

### Ischemic heart disease

Long-term PM₂.₅ exposure is associated with IHD mortality (RR 1.16 per 10 µg/m³) [11] and with incident coronary events [22, 44], and short-term exposure with acute myocardial infarction [20]. The mechanisms described above — endothelial dysfunction, accelerated atherosclerosis [69, 70], plaque inflammation and a prothrombotic state — provide biological plausibility, and controlled exposure of patients with coronary disease to diesel exhaust increased exercise-induced myocardial ischemia [49]. Air pollution thus appears to act both as a long-term contributor to atherosclerosis and as a short-term trigger of acute coronary events [74].

### Stroke

Long-term PM₂.₅ exposure is associated with stroke mortality (RR 1.11 per 10 µg/m³) [11], and short-term exposures to PM₂.₅, PM₁₀, NO₂, SO₂ and CO are associated with stroke hospitalization or death, with the strongest associations on the day of exposure [25].

### Hypertension

In a meta-analysis, both short- and long-term exposures to several pollutants, including PM and NO₂, were associated with a higher prevalence of hypertension [26]. The mechanisms most likely to connect exposure to clinical hypertension are sympathetic activation [65], acute vasoconstriction and endothelial dysfunction [23, 48], low-grade inflammation [61] and, on the basis of animal data, interaction with the RAAS [67]. Supporting a causal link, randomized trials of portable high-efficiency particulate air (HEPA) filtration have reported modest reductions in systolic blood pressure [75, 76].

Sympathetic overactivity is also central to resistant hypertension, particularly in patients with chronic kidney disease, in whom renal denervation has been shown to lower blood pressure safely and effectively, as summarized in the recent review by Iliakis et al. [77]. Whether chronic pollution-related sympathetic activation contributes to difficult-to-control hypertension, or modifies the response to renal denervation, has not been studied and represents a testable clinical question.

### Heart failure

Short-term increases in PM₂.₅, PM₁₀, NO₂, SO₂ and CO are associated with HF hospitalization or death (e.g., + 2.12% per 10 µg/m³ PM₂.₅) [21]. Proposed mechanisms include increased afterload from vasoconstriction and higher blood pressure, myocardial ischemia and, in animal models, myocardial oxidative stress and mitochondrial dysfunction [59, 62]. Given the large and growing global burden of HF [37], long-term cohort studies of incident HF in high-exposure LMIC settings are a priority.

### Arrhythmias

Short-term exposure to PM and several gaseous pollutants has been associated with arrhythmia hospitalization and outpatient visits in meta-analysis [78], and a nationwide Korean cohort reported an association between long-term PM₂.₅ exposure and incident atrial fibrillation [79]. Reduced HRV provides a plausible mechanism [65], but the evidence is less consistent than for IHD and is affected by variation in arrhythmia ascertainment.

### Vascular disease and arterial stiffness

Long-term PM₂.₅ exposure is associated with progression of subclinical atherosclerosis [69, 70], and controlled exposure to cookstove smoke acutely altered measures of arterial stiffness and central blood pressure [80]. Evidence for peripheral artery disease and venous thromboembolism is more limited [47].

## Susceptible populations and health inequities

Susceptibility reflects both biological vulnerability and differential exposure. Older adults and people with pre-existing CVD, diabetes or chronic lung disease show larger relative or absolute increases in risk [3, 7]. In the U.S. Medicare cohort, associations between PM₂.₅ and mortality were stronger among men, Black beneficiaries and those eligible for Medicaid (a marker of low income) [27].

Exposure is also unequally distributed. In the United States, people of color are exposed to higher PM₂.₅ concentrations from nearly all major emission sectors, regardless of income [81]. A global review found consistent evidence that lower socioeconomic position is associated with higher exposure in North America, whereas findings from Europe, Asia and other regions were more mixed [14]; in China, population exposure to PM varies with regional socioeconomic development [82]. At the global level, the age-standardized burden of PM-attributable CVD is highest in low- and low-middle-SDI regions [5]. In India, 1.24 million deaths in 2017 were attributed to air pollution, and 76.8% of the population was exposed to annual ambient PM₂.₅ above the national standard of 40 µg/m³ [83]. In such settings, high ambient concentrations coincide with household exposure from solid-fuel use, which is concentrated among rural and low-income households [16, 31].

## Molecular and multi-omics signatures of exposure

Omics studies can reveal pathways activated by exposure and generate candidate biomarkers, but most are small, cross-sectional or experimental, rarely replicated and not linked to clinical outcomes. The findings below should therefore be regarded as hypothesis-generating rather than as established clinical biomarkers or therapeutic targets.

### Transcriptomics

In human AC16 cardiomyocytes treated with PM₂.₅, genome-wide expression profiling identified 472 differentially expressed genes, with enrichment of cytokine–cytokine receptor interaction, NF-κB and chemokine signaling pathways [84]. In human umbilical vein endothelial cells, PM₂.₅ up-regulated genes involved in oxidative-stress and endoplasmic reticulum stress responses, including heme oxygenase-1 (*HMOX1*) [85]; in vascular tissue, PM exposure has been linked to increased expression of adhesion molecules and reduced endothelial nitric oxide synthase [86]. In bone marrow-derived endothelial progenitor cells from mice exposed to concentrated ambient PM₂.₅, 166 genes were differentially expressed, including reduced expression of *Tek* (Tie2) and *Cxcr3* and increased expression of *Ccl5*, consistent with impaired progenitor-cell maturation and mobilization [87, 88]. In humans, long-term residential PM₂.₅ exposure was associated with changes in peripheral blood gene expression in pathways related to cell signaling and innate immunity in two Dutch cohorts, with directionally consistent results in exposure-discordant monozygotic twin pairs, which reduces confounding by genetic background [89].

### Proteomics and lipidomics

Circulating protein changes associated with PM exposure in humans include higher CRP, IL-6 and fibrinogen [56, 57] and markers of endothelial injury and platelet activation [58]; broader biomarker panels have been reviewed elsewhere [90]. Exposure has also been associated with adverse lipid profiles, including higher low-density lipoprotein cholesterol, oxidative modification of LDL and impaired high-density lipoprotein anti-oxidant function, which may promote foam-cell formation [70, 74]. Untargeted proteomic and lipidomic studies remain few and small, and none has yet shown that a pollution-related molecular profile improves cardiovascular risk prediction.

### Epigenetics

Exposure to traffic particles has been associated with rapid changes in blood DNA methylation, including hypomethylation of repetitive elements, in older men [91]. Such changes may represent a mechanism by which exposures have lasting effects, but causality, tissue specificity and persistence remain uncertain [15].

### MicroRNAs

MicroRNAs regulate inflammation, endothelial function and atherosclerosis and have been proposed as mediators and biomarkers of pollution effects [92]. In older men of the Normative Aging Study, short-term PM₂.₅ exposure was associated with altered — predominantly lower — blood expression of several inflammation- and endothelium-related miRNAs, including miR-21, miR-126, miR-146a and miR-155 [93]. In experimental models, PM₂.₅ increased miR-155-5p in airway epithelial cell-derived exosomes, which promoted macrophage inflammatory responses [94], and in patients with coronary artery disease, ambient O₃ was associated with changes in extracellular-vesicle miRNAs, including miR-150 [95]. The direction of change differs between studies, tissues and exposure windows, and none of these miRNAs has been validated as a clinical biomarker or therapeutic target for pollution-related CVD (Table 5).

**Table 5:** MicroRNAs studied in relation to air pollution and cardiovascular disease: evidence type and validation status

miRNA	Exposure	Study type / model	Reported change	Proposed role	Validation status	Ref.
miR-21	PM₂.₅ (short-term)	Human cohort (older men; blood)	Lower expression	Inflammation, endothelial function	Exploratory; direction inconsistent across studies	[93]
miR-155	PM₂.₅; O₃	Human cohort (blood); cell/exosome models	Lower in blood (cohort); higher in epithelial exosomes (experimental)	Macrophage activation, cytokine production	Experimental; not clinically validated	[93, 94]
miR-126	PM₂.₅ (short-term)	Human cohort (older men; blood)	Lower expression	Endothelial integrity and repair	Exploratory; not clinically validated	[93]
miR-150	O₃	Patients with CAD (extracellular vesicles)	Altered expression	Inflammatory regulation	Exploratory; single study	[95]

CAD, coronary artery disease; miRNA, microRNA; O₃, ozone; PM₂.₅, fine particulate matter. None of these miRNAs is currently validated as a clinical biomarker or therapeutic target for pollution-related cardiovascular disease

## Current knowledge gaps and future directions

Several gaps limit the translation of current evidence into individualized prevention. First, most multi-omics studies are cross-sectional or experimental, use single time points, come from high-income countries and lack clinical endpoints, so it is unknown whether pollution-related molecular signatures predict cardiovascular events. Second, AI and machine-learning methods have improved exposure assessment — for example, ensemble models that estimate daily PM₂.₅ at 1-km resolution [96] — but their use for individual cardiovascular risk prediction has not been validated prospectively, and models trained on high-income data may perform poorly in LMICs. Third, precision-medicine approaches have not yet identified any genetic or molecular profile that should change the management of a patient exposed to high pollution.

We propose two research directions that the existing literature has not addressed. (1) **Prospective exposure–omics–outcome cohorts in high-exposure LMIC settings**, combining personal or high-resolution modelled exposure with repeated multi-omics sampling (proteome, methylome, miRNA) and adjudicated cardiovascular endpoints, to test whether a pollution-responsive molecular signature adds prognostic information to conventional risk scores and exposure alone. Such cohorts would also allow MR and gene–environment interaction analyses of candidate mediators. (2) **Pollution-stratified mechanistic trials of existing therapies.** No trial has tested whether interventions targeting the pathways activated by pollution — for example, anti-inflammatory therapy targeting IL-1β or IL-6 [64, 97], RAAS or aldosterone-targeted therapy [98] or sympathetic modulation [77] — attenuate pollution-related increases in blood pressure, inflammatory markers or cardiovascular events. Pragmatic trials in highly exposed populations, with exposure pre-specified as an effect modifier, could address this question. In both directions, AI-based exposure and risk models should be evaluated prospectively, including for performance and fairness in LMIC populations.

## Public health implications and preventive strategies

The gap between scientific evidence and regulation remains large. WHO lowered its annual PM₂.₅ guideline from 10 µg/m³ (2005) to 5 µg/m³ in 2021 [9], but many national standards are considerably higher — for example, India’s annual PM₂.₅ standard is 40 µg/m³ [83]. Because the cardiovascular benefits of cleaner air accrue across the population, and because disadvantaged groups have the highest exposures, regulatory action is also a matter of health equity [14, 99]. Preventive measures differ substantially in the strength of their evidence (Fig. 4).

**Fig. 4:**
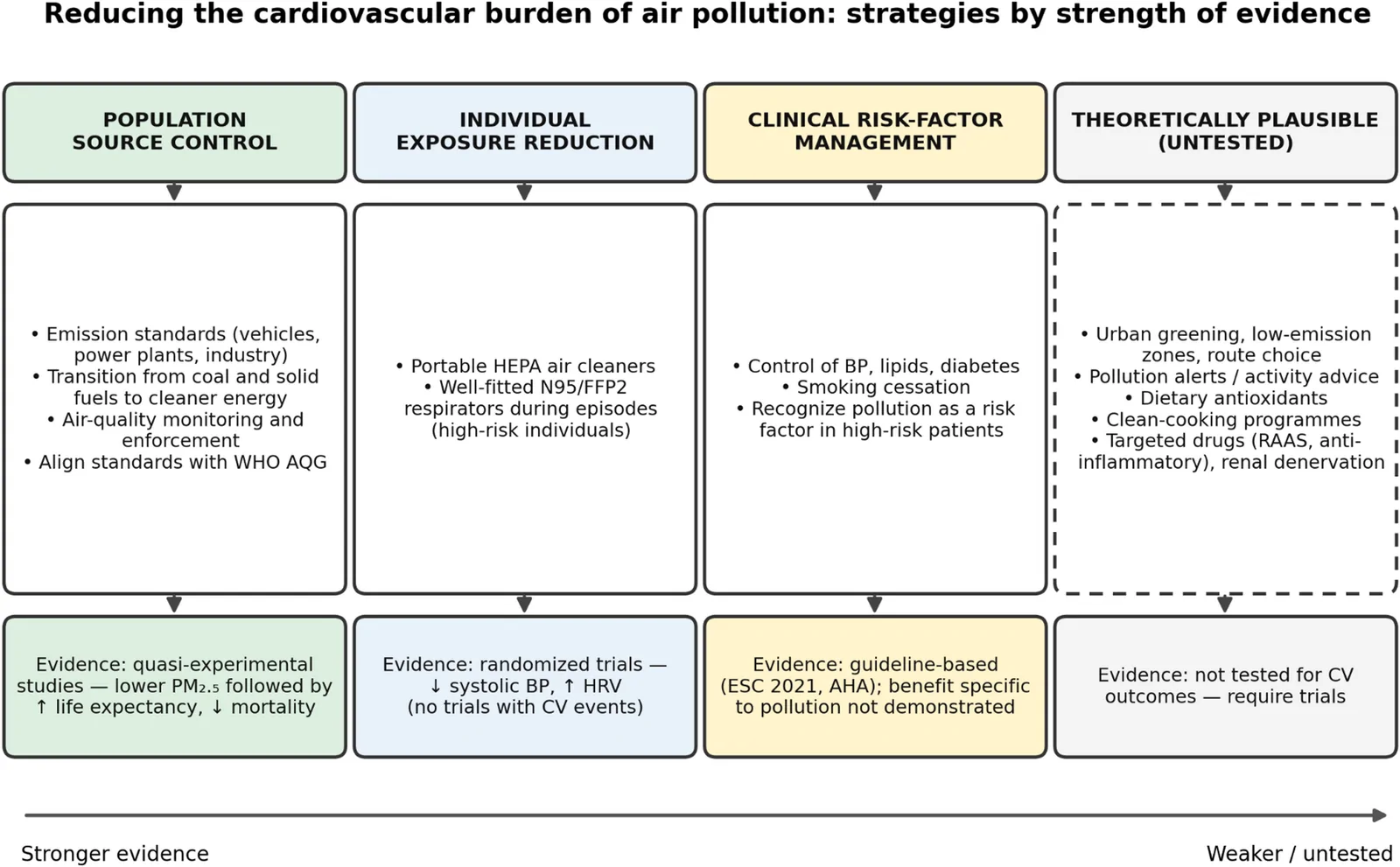
Strategies to reduce the cardiovascular burden of air pollution, grouped by strength of evidence: population-level source control (supported by quasi-experimental evidence on mortality); individual exposure reduction (supported by randomized trials on intermediate endpoints); clinical risk-factor management (guideline-based); and theoretically plausible measures that have not been tested for cardiovascular outcomes. Based on [51, 75, 99– 101]

### Evidence-based measures

#### Population-level source control

The strongest evidence that reducing pollution benefits cardiovascular health comes from quasi-experimental studies. Across U.S. metropolitan areas, a 10 µg/m³ reduction in PM₂.₅ was associated with an increase in life expectancy of approximately 0.61 years [51], and reductions in PM₂.₅ in the Six Cities cohort were followed by lower mortality [42]. Measures supported by this evidence include stricter emission standards for vehicles, power plants and industry, transition from coal and other solid fuels to cleaner energy, and enforcement supported by transparent air-quality monitoring [9, 99].

#### Individual exposure reduction

Randomized trials show that portable HEPA air cleaners reduce indoor PM₂.₅ and modestly lower systolic blood pressure [75, 76], and that wearing an efficient face mask during high-pollution periods improved blood pressure and HRV in patients with coronary heart disease [102]. The American Heart Association considers portable air cleaners and N95 respirators reasonable options for high-risk individuals during periods of high pollution [100]. These trials used intermediate endpoints, and no trial has yet shown a reduction in cardiovascular events

#### Clinical risk-factor management

Current guidelines recognize air pollution as a cardiovascular risk factor [99, 101]. Optimal control of blood pressure, lipids and diabetes, and smoking cessation, are evidence-based for cardiovascular prevention in general and are particularly important in people with high exposure, although benefit specifically against pollution-related risk has not been demonstrated.

### Theoretically plausible measures requiring evaluation

Other measures are biologically plausible but have not been tested for cardiovascular outcomes: urban greening, low-emission zones and active-travel route choice; air-quality alert systems and advice to limit outdoor exertion on high-pollution days; dietary antioxidants and other nutritional supplements; and clean-cooking programmes in households using solid fuels, which reduce exposure but have limited trial evidence for cardiovascular endpoints [16, 100]. Pharmacological targeting of pathways activated by pollution is a further hypothesis. For example, experimental data suggest that PM interacts with angiotensin II signaling [67], and aldosterone synthase inhibitors lowered systolic blood pressure in resistant hypertension in a meta-analysis of four randomized trials (776 participants), although with more adverse events than placebo [98]. Whether such agents, or renal denervation [77], provide particular benefit in patients with high pollution exposure is unknown and should be tested rather than assumed.

## Limitations

This review has limitations. As a narrative review, it did not include a formal risk-of-bias assessment or meta-analysis, and study selection may have been influenced by the authors’ judgement and the restriction to English-language publications. The global burden estimates cited are model-based and depend on assumed exposure–response functions and counterfactual concentrations. Most epidemiological evidence is observational and subject to residual confounding and exposure misclassification; evidence from LMICs, where exposures are highest, remains limited; and much of the mechanistic and omics evidence comes from cell and animal studies at exposure levels that may not reflect ambient human exposure.

## Conclusions

PM₂.₅ is a major, modifiable cardiovascular risk factor: it is associated with approximately 4.5 million cardiovascular deaths worldwide each year, and the combined epidemiological, controlled-exposure and quasi-experimental evidence supports a causal relationship with ischemic heart disease, stroke, heart failure and other cardiovascular outcomes. For clinicians, the main message is that air pollution exposure should be considered in cardiovascular risk assessment and prevention, particularly for high-risk patients. Important uncertainties remain, including risks at very low concentrations, the relative toxicity of particle components and gaseous pollutants, and whether individual exposure-reduction measures reduce clinical events. Priorities for research are prospective exposure–omics–outcome studies in LMICs, causal-inference designs such as MR, and trials of exposure-reduction and targeted therapeutic strategies in highly exposed populations.

## Data Availability

No datasets were generated or analysed during the current study.
